# Quantum hydrodynamics of a single particle

**DOI:** 10.1038/s41377-020-0324-x

**Published:** 2020-05-13

**Authors:** Daniel Gustavo Suárez-Forero, Vincenzo Ardizzone, Saimon Filipe Covre da Silva, Marcus Reindl, Antonio Fieramosca, Laura Polimeno, Milena De Giorgi, Lorenzo Dominici, Loren N. Pfeiffer, Giuseppe Gigli, Dario Ballarini, Fabrice Laussy, Armando Rastelli, Daniele Sanvitto

**Affiliations:** 1 CNR NANOTEC, Institute of Nanotechnology, Campus Ecotekne, Via Monteroni, 73100 Lecce, Italy; 2 Dipartimento di Ingegneria dell’Innovazione, Università del Salento, Campus Ecotekne, via Monteroni, 73100 Lecce, Italy; 3 Institute of Semiconductor and Solid State Physics, Johannes Kepler University, Altenbergerstr. 69, Linz, 4040 Austria; 4 Dipartimento di Matematica e Fisica E. De Giorgi, Università del Salento, Campus Ecotekne, via Monteroni, Lecce, 73100 Italy; 5 PRISM, Princeton Institute for the Science and Technology of Materials, Princeton University, Princeton, NJ 08540 USA; 6 Faculty of Science and Engineering, University of Wolverhampton, Wulfruna Street, Wolverhampton, WV1 1LY UK; 7 Russian Quantum Center, Novaya 100, 143025 Skolkovo, Moscow Region, Russia

**Keywords:** Polaritons, Quantum optics, Polaritons, Quantum optics

## Abstract

Semiconductor devices are strong competitors in the race for the development of quantum computational systems. In this work, we interface two semiconductor building blocks of different dimensionalities with complementary properties: (1) a quantum dot hosting a single exciton and acting as a nearly ideal single-photon emitter and (2) a quantum well in a 2D microcavity sustaining polaritons, which are known for their strong interactions and unique hydrodynamic properties, including ultrafast real-time monitoring of their propagation and phase mapping. In the present experiment, we can thus observe how the injected single particles propagate and evolve inside the microcavity, giving rise to hydrodynamic features typical of macroscopic systems despite their genuine intrinsic quantum nature. In the presence of a structural defect, we observe the celebrated quantum interference of a single particle that produces fringes reminiscent of wave propagation. While this behavior could be theoretically expected, our imaging of such an interference pattern, together with a measurement of antibunching, constitutes the first demonstration of spatial mapping of the self-interference of a single quantum particle impinging on an obstacle.

## Watching a single particle behave like a wave

Pioneering experimental observations of the quantum interference of single particles vividly illustrate wave-particle duality, and point the way towards manipulating individual excitations for quantum computing. An international team including Vincenzo Ardizzone and co-workers at CNR Nanotec in Lecce, Italy, generated single photons by directing a pulsed laser onto a nano-sized crystal called a quantum dot. These photons then interacted with excitons in a semiconductor cavity to produce hybrid particles called polaritons, which show great promise as switchable quantum bits (qubits) in future computers. Most interestingly, the researchers observed a single-polariton scattering off a defect in the cavity, leaving wave-like fringes where the particle’s incoming wavefunction interfered with the scattered parts. This observation represents the first two-dimensional mapping of wave-particle duality for a single quantum particle, and is reminiscent of the famous double-slit experiments.

## Introduction

The generation, manipulation and detection of on-chip single photons is key to the development of photonic-based quantum information technologies^1^. Integrated optics (IO) devices working in the single-particle regime will enable deployment of quantum information processing for both fundamental research and technological applications. IO chips should provide qubit generation, processing and readout. For instance, qubit generation can be implemented by using semiconductor quantum dots (QDs)^2,3^ or parametric sources^4,5^. Superconducting single-photon detectors^6^ seem to be among the most promising candidates to date for integrated qubit detection^7,8^. Most of the optical circuits used for quantum information thus far rely on the linear properties of single-photon propagation^9^ or on the optical nonlinearities of *χ*^2^ or *χ*^3^ materials^10–12^. By combining these elements, several functionalities, such as quantum logic gates^13,14^, boson sampling^15^, quantum interference or quantum metrology^16^, have been demonstrated. However, present schemes for single qubit manipulation face real challenges, relying on complex cascades of linear optical elements or on weak nonlinear susceptibilities requiring long interaction paths. These features could result in severe limitations on the scalability and miniaturization of future devices.

Microcavity polaritons, hybrid light-matter quasiparticles emerging from the strong coupling between a cavity mode and an excitonic transition, could represent a promising alternative to achieving quantum information processing in integrated optical circuits^17^. Their intrinsically interacting nature, inherited from their excitonic component, together with their long coherence time, inherited from their light component, make them strong candidates for performing nonlinear logic operations without losing information^18,19^. While collective mesoscopic phenomena involving microcavity polaritons—such as polariton lasing, superfluidity or optical parametric oscillation—have been extensively studied^20^, there has been little exploration of their quantum, few-particle limit, i.e., non-Gaussian polariton states. The experimental demonstrations of polaritonic quantum behavior have been mainly limited to Gaussian mixtures at best^21–23^, with only recent progress towards the generation of non-Gaussian states, following the demonstration of polariton blockade^24–26^. Taking the entirely different approach of exciting polaritons with quantum light^27^, it was experimentally demonstrated that the creation and recombination of polaritons in a semiconductor microcavity can be achieved without damaging the quantum coherence of nonclassical states, which is a necessary condition for any quantum information processing^28^.

In this work, we demonstrate how one can use single photons emitted by an external semiconductor QD to generate, inject and propagate individual microcavity polaritons—a fundamental milestone for the development of future polariton quantum devices. Moreover, with this experiment, we show the ability to map the propagation of single polaritons in two-dimensional space during the propagation time, thus bringing down to the ultimate single-particle limit the prominent and remarkable propagation of a polariton fluid. This is the first step towards several single-particle configurations in a solid-state, integrable setup manipulating highly interacting single-polariton qubits. In our case, still with a single polariton, by imaging its propagation across an obstacle acting as a scattering center, we can observe the wave-like interferences produced by what is otherwise one polariton alone. This observation represents an alternative version of the double-slit experiment, directly demonstrating the wave-particle duality for individual microcavity polaritons^29,30^. While wave-particle duality is expected for single quantum particles, our experiment represents the first 2D mapping of such behavior. In our case, instead of a double slit or single particle splitting, we observe the interference effect on multiple path-scattering propagation of a single particle impinging on an obstacle smaller than the wavepacket size, providing direct imaging of the wave-particle duality of these light-matter excitations^31^.

## Results

**Fig. 1:**
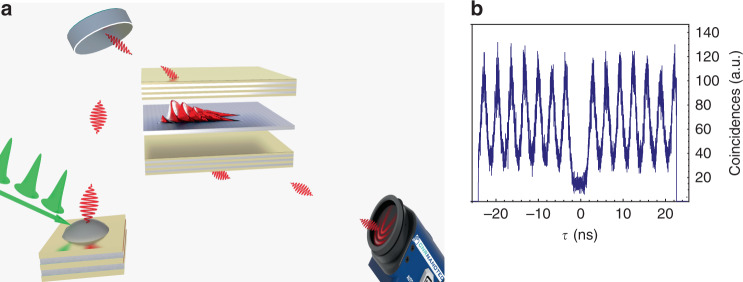
Generation, injection and detection of single polaritons; second-order correlation function for the pumping photons. **a** Schematic of the experiment: a pulsed laser pumps a QD to generate single photons that are injected inside a semiconductor microcavity. An image of the single polariton propagation is acquired with an EMCCD. **b** Second-order correlation function of QD emission when multiplexing the pump pulse rate to 320MHz; The antibunching value of *g*^(2)^(0) = 0.16 ± 0.05 is an unequivocal signature of single-photon emission from these QDs

A scheme of the experimental setup is shown in Fig. 1a. It is composed of three main parts: (i) generation of single photons, (ii) injection and propagation of single polaritons and (iii) detection. The imaging and spectroscopy experiments were performed in both reflection and transmission configurations. The latter required processing of the substrate to enable transmission and optimization of the signal intensity by increasing the single-photon emission rate. A more detailed representation of the experimental setup can be found in the Supplementary Material. Despite its conceptual simplicity, our hybrid approach that couples a single-photon source, providing qubits, to polaritons in a high-quality-factor microcavity, propagating as single particles, presents considerable technical difficulties. First, the use of a tuneable source of heralded single photons, i.e., spontaneous parametric down conversion (SPDC) in nonlinear crystals, must be ruled out. This kind of source would imply a heralded measurement that ensures that the propagation of single photons is measured, resulting in a very cumbersome implementation for common imaging systems. Additionally, the broad emission spectra of SPDC sources^5^ would be poorly coupled to the microcavity, whose high-quality factor is necessary to confine the quantum state long enough to sustain polariton propagation. We thus decided to use single QDs as a deterministic single-photon source with an emission linewidth compatible with the narrow polariton resonance. However, most common QD systems (e.g., InGaAs QDs grown with the Stranski-Krastanow method) have a typical emission range incompatible with GaAs/AlGaAs microcavities. We use therefore GaAs QDs produced by Al droplet etching, which have been recently shown to be nearly ideal single photon sources^32^ with *g*^(2)^(*τ* = 0) below 10^−4^. The choice of QDs as single-photon sources entails an additional obstacle to overcome: the necessity of keeping both systems, the QDs and microcavity, at cryogenic temperatures. Here, we demonstrate that these issues can be overcome by employing an alternative platform for the study of polariton systems in the single or few particle regime involving several qubits.

**Fig. 2:**
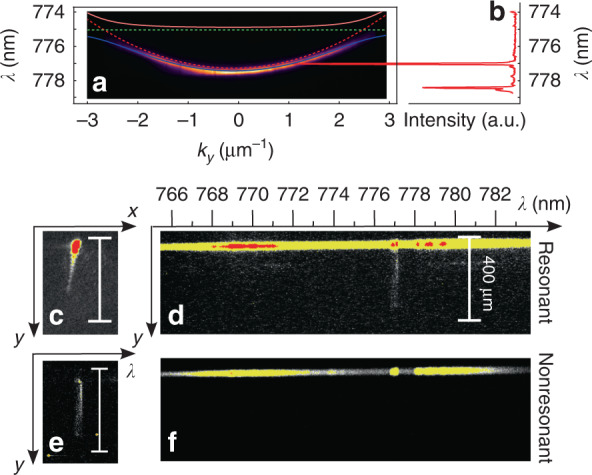
Single polariton propagation measured in reflection configuration. **a** Energy dispersion of the microcavity-quantum well system at the point of incidence of photons in the reflection configuration compared with the emission spectrum of the pumping QD, shown in (**b**). At *k*_*y*_ ≈ 1.1μm^−1^, the exciton energy is in resonance with the LPB, allowing resonant polariton injection into the microcavity; polaritons injected with this in-plane momentum propagate with a group velocity *v*_*g*_ ≈ 2.1µm/ps, as deduced in the Supplementary Material; **c** Real space image of the single polariton propagation. **d** Energy-resolved propagation in c, evidencing how only the QD exciton peak couples into the system and propagates. **e** To obtain a better image of the propagation, the noncoupled reflected light is blocked by a spatial filter; polariton propagation distances are measured up to ~400µm. **f** At a different microcavity-quantum well detuning, the QD exciton is not in resonance with the polariton dispersion, and indeed, no propagation is evidenced in this situation

In the following experiments, single photons are generated from GaAs QDs fabricated by Al droplet etching and embedded in a low-Q cavity consisting of a *λ/*2 layer of Al_0.4_Ga_0.6_As with QDs sandwiched between two Bragg mirrors made of 9 and 2 pairs of Al_0.95_Ga_0.05_As (67 nm thick) and Al_0.2_Ga_0.8_As (55 nm thick)^32,33^. The QDs are pumped with a pulsed laser either at *λ* = 415 nm with a pulse duration of ~30 fs or at 780 nm with a pulse duration of ~5 ps. Owing to a pair of cascaded Michelson and Morley interferometers, we can increase the laser repetition rate by a factor of four, from 80 to 320 MHz, to increase the number of photons per second (for more details on the photon-rate quadruplication, see the Supplementary Material). The QD size is optimized to obtain an emission wavelength of approximately 775 nm. The generated photons are coupled to a single mode fiber and then used to pump the polaritonic device, a *λ/*2 microcavity made of two Bragg mirrors of 40 and 32 pairs of Al_0.96_Ga_0.04_As (67 nm thick) and Al_0.2_Ga_0.8_As (55 nm thick) with a quantum well 7.2 nm wide embedded in the center. Both systems, the QDs and microcavity sample, are cooled down to cryogenic temperatures of 3.8 and 8.5 K, respectively. A comparison between the spectra of the microcavity and the quantum dot is shown in Fig. 2a,b. Despite the mentioned technical difficulty, we succeeded in growing QDs with a transition energy precisely in the energy range covered by the lower polariton branch (LPB) of the microcavity-quantum well system^34^. By carefully tuning the single-photon injection angle, the QD emission can be resonantly coupled to the LPB. The quantum nature of the light is tested by measuring the second-order correlation function *g*^(2)^(*τ* = 0) with a Hanbury Brown and Twiss setup (HBT, Fig. 1b), finding a value of *g*^(2)^(0) = 0.16 ± 0.05. The small residual difference from zero is attributed to the nonresonant excitation as well as the slow carrier relaxation in the QDs^35^. Real space images are obtained by an enhanced charge coupled device (EMCCD) camera coupled to a monochromator to allow energy-resolved measurements.

Figure 2c shows a real-space image of the microcavity surface under single-photon excitation. The image shows two main features: a bright saturated circular spot and a weaker elongated spot. We assign the former to the reflected uncoupled single photons and the latter to the resonantly excited single polaritons that propagate inside the microcavity due to their externally imparted in-plane momentum. To prove this, we acquire an energy-resolved image (see Fig. 2d). This image shows that among all the peaks in the QD emission, the neutral exciton peak resonant with the LPB is the only one able to couple to and propagate inside the sample, as evidenced by the faint but continuous vertical trace at the corresponding wavelength. The high Q factor of the cavity results in high energy selectivity and long propagation distances, close to 400 µm. To further prove that the propagation corresponds to resonant polariton injection, the sample is moved to a point at which the LPB is not resonant with the QD emission, as shown in Fig. 2f. In this case, no single polariton propagation is observed. By fitting the intensity profile of the propagating polaritons with an exponential decay and considering that single polaritons are injected with an in-plane momentum of 1.1 µm^−1^ (see Supplementary Material), we obtain a polariton lifetime of τ ∼ 25 ps, which is in agreement with the lifetime that can be deduced from the polariton linewidth.

**Fig. 3:**
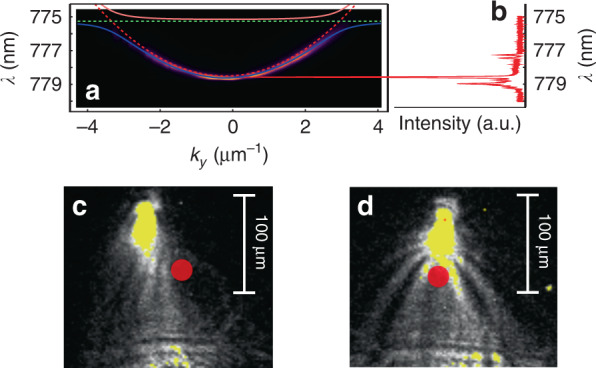
Single polariton propagation measured in transmission configuration. **a** Energy dispersion in the microcavity at the photon injection point in the transmission configuration compared with the emission spectrum of the selected QD, shown in panel b; note that the exciton energy of the QD coincides with the state of the LPB with in-plane momentum *k*_*y*_ ≈ 0.28 *µ*m^−1^; this allows resonant pumping of the microcavity with single photons from the QD; the second-order correlation function in Fig. 1b is precisely obtained for this QD emission spectrum; Single-polariton propagation obtained by matching the polariton dispersion in (**a**) with the single photons corresponding to (**b**). **c**, **d** Single-polariton propagation across a defect naturally occurring in the microcavity; an interference pattern appears due to the self-interference between the incoming wavefunction and its scattering against the defect; the red circle indicates the position of the structural defect

The reflection configuration is compelling for a proof-of-principle demonstration and as a first attempt to demonstrate the phenomenon, also allowing us to prove that only the single-photon excitonic peak couples to the microcavity and triggers propagation inside it, while the other peaks are fully reflected. However, in the reflection configuration, it is impossible to image the region around the point of injection. To obtain a more comprehensive picture of the single polariton propagation, we modify our setup to perform experiments in a transmission configuration. To do so, the polariton sample is processed by wet etching to remove the absorbing bulk GaAs substrate in selected regions and uncover the microcavity^36^. A comparison of a QD emission spectrum and the microcavity LPB dispersion corresponding to an etched region is shown in Fig. 3a,b. The microcavity dispersion (panel a) is measured in transmission. Again, a QD with an excitonic peak (in this case, a positively charged exciton) near the LPB is chosen to resonantly excite single polaritons. Moreover, to increase the amount of signal, the pulse repetition rate of the laser exciting the QD is quadruplicated, up to a final rate of 320 MHz. To carefully check the single-photon regime, we measure the second-order correlation function after each doubling of the pump repetition rate. The corresponding measurements, shown in Fig. 6 of the Supplementary Material, confirm that the QD always behaves as a single-photon emitter. After this optimization, the microcavity sample is pumped with approximately 140 k single photons per second. To decrease the total recombination time of the exciton to avoid temporal overlap of the generated photons, the pump wavelength is changed from 405 to 780 nm. Indeed, the QD decay time involves several processes, but in general, higher pump energies require more nonradiative processes, entailing longer decay times^37^. Although different QDs are used to pump the cavity in the reflection and transmission configurations, in all cases, a *g*^(2)^ measurement such as that shown in Fig. 1b is obtained.

Two cases were considered for the single polariton propagation in the transmission configuration: “free propagation” along the microcavity (shown in Fig. 3c) and propagation in the presence of an obstacle (shown in Fig. 3d), given by a structural defect in the microcavity. The single-polariton propagation in Fig. 3c,d spreads slightly more than that in Fig. 2c–e. This is obtained by modifying the excitation beam divergence, making it easier to image defects in the cavity. The polariton group velocity, estimated through the dispersion relation, is *v*_g_ ≈ 0.3 µm ps^−1^. For a more complete description of the deduction of the group velocity, we refer the reader to the Supplementary Material.

**Fig. 4:**
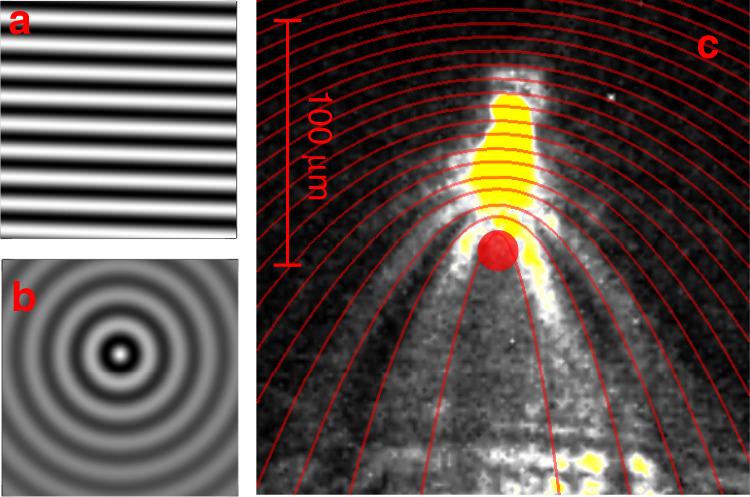
Self-interference of individual polaritons. **a** Numerical spatial distribution of the electric field of an incoming plane wave, and (**b**) that for a circular wave, as it could be used to model the light scattered from a point defect in the microcavity. **c** Experimental density map from Fig. 3d with superimposed numerical simulations of the single-polariton self-interference; red lines are contour lines with the same intensity calculated by making the wavefront interfere with the circular wave in b; the numerical simulations are obtained by assuming an incoming polariton with in-plane momentum *k* = 0.28 µm^−1^, as in the experiment

The second case, shown in Fig. 3d, is even more interesting. It shows the same single-polariton propagation but now across an obstacle formed by a structural defect, highlighted by the red dot. The defect scatters the incoming single polaritons and substantially modifies the propagation pattern. Clearly, some interference is formed, as observed in Fig. 3d. While this would be the expected pattern for a conventional polariton fluid passing an obstacle, here, it must be borne in mind that in the conditions of our experiment, these fringes arise from the integration of photons emitted by polaritons that each travel alone in the microcavity, given that they have been injected into there by a strongly antibunched single-photon source with a repetition rate-to-polariton lifetime ratio such that each polariton is separated from the previous and next polaritons by more than 285 thousand times its lifetime. In other words, the interference patterns in Fig. 3d and the propagation in Fig. 3c are obtained by integrating several single-polariton propagations. There is no interference between subsequent photons spontaneously emitted by polaritons because only one polariton at a time is present in the microcavity. It is therefore surprising, from a classical perspective, that one polariton would simultaneously propagate through several distinct trajectories, as is required to produce destructive interference. This is a variation of the famous double-slit experiment, in which the wave-like aspect is more fully manifested owing to the possibility of mapping the polariton field everywhere in the real plane. Unlike the plethora of similar experiments performed with a screen at the end of the propagation^38–43^, in our case, photons are emitted from polaritons by spontaneous emission, and since this follows an exponential law, they have the same probability of being emitted at any time of their propagation. In other words, polaritons can provide a full mapping of their spatial dynamics. In our case, the interference pattern is simply explained by the interference between a plane wave, representing the incoming single-polariton (Fig. 4a), and a spherical wave, representing the scattered polariton (Fig. 4b). The sum of these two field amplitudes provides the interference pattern that we observe with excellent quantitative agreement, as shown in Fig. 4c as red lines on top of the experimental background. We note that the experimental interference pattern in Fig. 4 is consistent with a point-like defect scattering the incoming single-polariton plane wave. By point-like defect, we mean that its cross-section is smaller than the polariton wavelength, thus making the defect shape irrelevant for the effects measured here. In our case, the in-plane wavelength of the moving polariton can be estimated to be *λ*_//_ = 2*π/k* ≈ 20 µm. To confirm that the defect considered here is point-like, we simulated several interference patterns corresponding to various defect radii *r* ranging from *r* ≪ *λ*_//_ to *r* = *λ*_//_; see Fig. 4 in the Supplementary Material. It is evident from these simulations that when the defect cross-section becomes relevant, the pattern shows high-order interference features with several phase jumps giving rise to straight discontinuities across the main interference fringes. None of these features are present in our experimental data, supporting the hypothesis of a point-like defect. Moreover, the same model reproduces the data of Fig. 3c in which the defect is away from the main propagation axis of the single-polariton; see Fig. 5 in the Supplementary Material.

## Discussion

The apparent paradox of the double-slit experiment is currently familiar in quantum theory. Feynman called it “the only mystery of quantum mechanics”^30^. Nevertheless, its implications and deep underlying meaning still captivate one’s imagination. The polariton platform could contribute to this foundational and fundamental issue. The wonders of quantum mechanics in our spatially mapped version of the double-slit experiment are even more salient based on the fact that the fringes are visible ahead of the obstacle, meaning that even though the single-polariton is not yet supposed to know that an obstacle lays ahead, its plane-wave forward motion probability amplitude is already interfering with its backward-scattered spherical motion probability amplitude, thus suppressing the spontaneous emission from the polariton at any point of destructive interference even ahead of the obstacle.

Even though the wave-particle duality for single particles is expected in quantum mechanics, access to the full spatial profile of the field opens new perspectives for deeper investigations of this key phenomenon that, more than any other, questions the nature of physical reality. This makes the platform, for instance, an interesting testbed for Wheeler’s delayed-choice experiment and its variations^44^ that question whether an observation can be conditioned on the past history of the particle. We have already touched upon how, in an interesting twist, the future of the particle is brought into question in our configuration. One could stretch the “particle” character of the polariton by imposing tighter constraints on its wavepacket size and localization and/or passing it through better designed potentials. This platform should also be feasible for exploring the violation of causal time-ordering^45^ in a quantum polariton switch. Also exciting is the possibility of involving multiple polaritons and studying nonlocal effects. In all cases, with the possibility of reconstruction of the full polariton-field wavefunction, we expect that our first demonstration of a propagating single polariton will give access to a wide range of fundamental quantum experiments in integrated optics.

In conclusion, we have demonstrated conversion of single photons from a semiconductor QD into propagating 2D microcavity polaritons by resonant injection into a semiconductor planar microcavity, and we have observed their propagation in a still minimally controlled environment, i.e., in the presence or absence of an obstacle. The observation of single-polariton propagation is a step towards the design and implementation of several single-polariton devices. At a fundamental level, we report the first observation of a polariton fluid that consists of a single particle and we confirm its wave-particle duality by observing fringes that result from wave interference for states that each consist of a single polariton. In contrast to the numerous earlier reports on one of the most important and far-reaching experiments of physics—the double-slit experiment—we have been able to provide the interference pattern in the full region of space where the phenomenon occurs. Further investigations of this phenomenon could allow us to better understand the fundamental aspects and ontological meaning of quantum theory at large. From an application point of view, the fact that both the QD and polaritons are based on the same material combination opens a route to fully integrated solutions, where polaritons may mediate the interaction of photonic qubits emitted by QDs.

## Methods

### Microcavity Sample

We use a *λ/*2 cavity embedded between two DBRs formed by 40 and 32 pairs of Al_0.96_Ga_0.04_As (67 nm thick) and Al_0.2_Ga0_0.8_As (55 nm thick), respectively. A 7.2 nm wide GaAs quantum well is placed in the center of the cavity at the maximum of the electric field. The sample substrate has been partially removed by wet etching to measure polariton propagation in the transmission geometry. The wet etching process has been carefully calibrated to selectively attack the substrate, and the number of pairs of the bottom DBR is not modified.

### QD Sample

GaAs QDs are fabricated by Al droplet etching and embedded in a low-Q cavity formed by a *λ/*2 layer that embeds the QDs between two DBRs formed by 9 and 2 pairs of Al_0.95_Ga_0.05_As and Al_0.2_Ga_0.8_As with thicknesses of 67 and 55 nm, respectively.

### Experimental realization

Both samples are kept at cryogenic temperatures in two different cryostats at 3.8 K for the QDs and 8.5 K for the microcavity. In the reflection configuration, the QD sample is pumped with a fs pulsed laser at 405 nm with a repetition rate of 80 MHz. In the transmission configuration, the excitation is performed with a 780 nm ps pulsed laser, which has been multiplexed by using a cascade of Michelson and Morley interferometers to obtain a final repetition rate of 320 MHz. The emission from the QD is collected in single-mode fiber optics and used to pump the microcavity sample in a configuration that allows fine control of the in-plane linear momentum. For the detection, an image of the propagation plane is reconstructed in an enhanced charge coupled device (EMCCD).

## Supplementary information

Supplemental Material

## References

[1] Won R (2019). Integrated solution for quantum technologies. Nat. Photonics.

[2] Huber D (2018). Semiconductor quantum dots as an ideal source of polarization-entangled photon pairs on-demand: a review. J. Opt..

[3] Elshaari AW (2017). On-chip single photon filtering and multiplexing in hybrid quantum photonic circuits. Nat. Commun..

[4] Kwiat PG (1999). Ultrabright source of polarization-entangled photons. Phys. Rev. A.

[5] Fedrizzi A (2007). A wavelength-tunable fiber-coupled source of narrowband entangled photons. Opt. Express.

[6] You LX (2017). Superconducting nanowire single-photon detector on dielectric optical films for visible and near infrared wavelengths. Superconductor Sci. Technol..

[7] Gourgues R (2019). Controlled integration of selected detectors and emitters in photonic integrated circuits. Opt. Express.

[8] Schwartz M (2018). Fully on-chip single-photon Hanbury-Brown and Twiss experiment on a monolithic semiconductor-superconductor platform. Nano Lett..

[9] Carolan J (2015). Universal linear optics. Science.

[10] Luo KH (2019). Nonlinear integrated quantum electro-optic circuits. Sci. Adv..

[11] Moss DJ (2013). New CMOS-compatible platforms based on silicon nitride and hydex for nonlinear optics. Nat. Photonics.

[12] Jin H (2014). On-chip generation and manipulation of entangled photons based on reconfigurable lithium-niobate waveguide circuits. Phys. Rev. Lett..

[13] Crespi A (2011). Integrated photonic quantum gates for polarization qubits. Nat. Commun..

[14] Politi A (2008). Silica-on-silicon waveguide quantum circuits. Science.

[15] Tillmann M (2013). Experimental Boson sampling. Nat. Photonics.

[16] Giovannetti V, Lloyd S, Maccone L (2011). Advances in quantum metrology. Nat. Photonics.

[17] Sanvitto D, Kena-Cohen S (2016). The road towards polaritonic devices. Nat. Mater..

[18] Ballarini D (2013). All-optical polariton transistor. Nat. Commun..

[19] Leyder C (2007). Interference of coherent polariton beams in microcavities: polarization-controlled optical gates. Phys. Rev. Lett..

[20] Carusotto I, Ciuti C (2013). Quantum fluids of light. Rev. Mod. Phys..

[21] Sassermann, M. et al. Quantum statistics of polariton parametric interactions. Preprint at https://arxiv.org/abs/1808.01127 (2018).

[22] Boulier T (2014). Polariton-generated intensity squeezing in semiconductor micropillars. Nat. Commun..

[23] Ardizzone V (2012). Bunching visibility of optical parametric emission in a semiconductor microcavity. Phys. Rev. B.

[24] Delteil A (2019). Towards polariton blockade of confined exciton-polaritons. Nat. Mater..

[25] Muñoz-Matutano G (2019). Emergence of quantum correlations from interacting fibre-cavity polaritons. Nat. Mater..

[26] Gerace D, Laussy F, Sanvitto D (2019). Quantum nonlinearities at the single-particle level. Nat. Mater..

[27] López Carreño JC (2015). Exciting polaritons with quantum light. Phys. Rev. Lett..

[28] Cuevas Á (2018). First observation of the quantized exciton-polariton field and effect of interactions on a single polariton. Sci. Adv..

[29] Kolenderski P (2015). Time-resolved double-slit interference pattern measurement with entangled photons. Sci. Rep..

[30] Feynman, R. P. *The Feynman Lectures on Physics—Volume 3: Quantum Mechanics* (Addison-Wesley, New York, 2010).

[31] Moreau PA (2019). Imaging bell-type nonlocal behavior. Sci. Adv..

[32] Schweickert L (2018). On-demand generation of background-free single photons from a solid-state source. Appl. Phys. Lett..

[33] Huber D (2018). Strain-Tunable GaAs quantum dot: a nearly dephasing-free source of entangled photon pairs on demand. Phys. Rev. Lett..

[34] Kavokin, A. V. et al. *Microcavities* 2nd edn. (Oxford Science, Oxford, 2008).

[35] Huber D (2017). Highly indistinguishable and strongly entangled photons from symmetric GaAs quantum dots. Nat. Commun..

[36] Moon EA, Lee JL, Yoo HM (1998). Selective wet etching of GaAs on Al_*x*_Ga_1-*x*_As for AlGaAs/InGaAs/AlGaAs pseudomorphic high electron mobility transistor. J. Appl. Phys..

[37] Reindl M (2019). Highly indistinguishable single photons from incoherently excited quantum dots. Phys. Rev. B.

[38] Aspect A, Grangier P (1987). Wave-particle duality for single photons. Hyperfine Interact..

[39] Tonomura A (1989). Demonstration of single-electron buildup of an interference pattern. Am. J. Phys..

[40] Dheur MC (2016). Single-plasmon interferences. Sci. Adv..

[41] Aspden RS, Padgett MJ, Spalding GC (2016). Video recording true single-photon double-slit interference. Am. J. Phys..

[42] Zeilinger A (1988). Single- and double-slit diffraction of neutrons. Rev. Mod. Phys..

[43] Arndt M (1999). Wave–particle duality of C_60_ molecules. Nature.

[44] Jacques V (2007). Experimental realization of Wheeler’s delayed-choice Gedanken experiment. Science.

[45] Goswami K (2018). Phys. Rev. Lett..

