# Supplementary material for: Quantum hydrodynamics of a single particle
Source: Light Sci Appl. 2020 May 13;9:85. doi: 10.1038/s41377-020-0324-x (PMC7221079; doi:10.1038/s41377-020-0324-x)
Supplement: Supplementary file 1 — Supplemental Material [file 41377_2020_324_MOESM1_ESM.pdf]

Supplementary Material: Quantum hydrodynamics of a single particle

Daniel Gustavo Suárez-Forero,[1,2] Vincenzo Ardizzone,[1,*] Saimon Filipe Covre da Silva,[3] Marcus Reindl,[3] Antonio Fieramosca,[1,4] Laura Polimeno,[1,4] Milena De Giorgi,[1] Lorenzo Dominici,[1] Loren N. Pfeiffer,[5] Giuseppe Gigli,[4] Dario Ballarini,[1] Fabrice Laussy,[6,7] Armando Rastelli,[3] and Daniele Sanvitto[1,†]

[1] *CNR NANOTEC, Institute of Nanotechnology, Via Monteroni, 73100 Lecce, Italy*
[2] *Dipartimento di Ingegneria dell'Innovazione, Università del Salento, via per Monteroni, km 1, 73100 Lecce, Italy*
[3] *Institute of Semiconductor and Solid State Physics, Johannes Kepler University, Linz, Altenbergerstr. 69, 4040, Austria*
[4] *Dipartimento di Fisica, Università del Salento, Strada Provinciale Lecce-Monteroni, Campus Ecotekne, Lecce 73100, Italy*
[5] *PRISM, Princeton Institute for the Science and Technology of Materials, Princeton Unviversity, Princeton, NJ 08540*
[6] *Faculty of Science and Engineering, University of Wolverhampton, Wulfruna Street, Wolverhampton WV1 1LY, UK.*
[7] *Russian Quantum Center, Novaya 100, 143025 Skolkovo, Moscow Region, Russia*

DEDUCTION OF THE POLARITON'S GROUP VELOCITY

By using a model of coupled oscillators, it is possible to fit the measured dispersion in order to obtain an analytical expression [1]. From the expression of $\omega(k)$, it is possible to obtain the group velocity as $\frac{d\omega}{dk}$. Fig. S1 shows the plot of the analytical expression found for the LPB (solid blue), UPB (solid pink), bare exciton (dashed green) and cavity mode (dashed red) for the reflection configuration shown in Fig. 2 of the main text. By changing units of the dispersion, in order to have the angular frequency ω as function of momentum k, and taking the first derivative, the analytical expression for the group velocity is obtained. In this case the in-plane momentum of the incoming photons is found to be $\approx 1.1\ \mu m^{-1}$, that corresponds to a group velocity of $\approx 2.1\ \mu m/ps$.

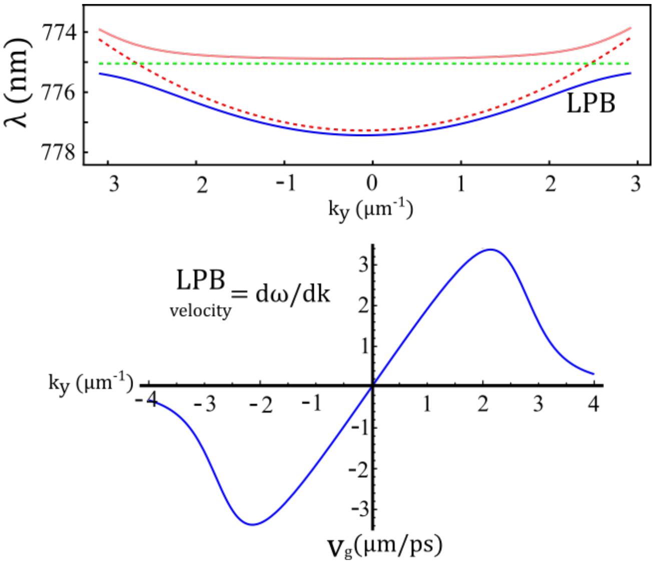

FIG. S1: Upper panel: analytical fit of the dispersion shown in figure 2 of the main text. As explained, the QD exciton's energy is in resonance with the LPB at $k_y \approx 1.1\ \mu m^{-1}$. Lower panel: group velocity for the fitted LPB.

EXPONENTIAL DECAY AND LIFETIME

Panels a and b of figure 2 of the main text show the wavelength and in-plane momentum of the injected particle (777.0 nm at $k_y = 1.1\ \mu\text{m}^{-1}$). As illustrated in last section, from the fitting with a theoretical model, the deduced group velocity is $\approx 2.1\mu m/ps$.

On the other hand, by making a decay profile of figure 2c of the main text, it is possible to get a decay length by doing an exponential fitting. As shown in figure S2, this length is measured to be 52 μm.

These two quantities give an estimation of the lifetime of $\tau_t = l_{decay}/v_g \approx 25$ ps, a value in agreement with the lifetime measured from the dispersion linewidth.

PULSE FREQUENCY MULTIPLICATION

The laser pulse frequency was quadruplicated in order to have enough signal to make the image of the propagation. This was done by using two Michelson and Morley interferometers with a delay of 6 ns (180 cm) in the first one and 3 ns (90 cm) in the second one. The main limitation in the frequency multiplication is given by the time resolution of the detection: the antibunching should always be measurable in order to guarantee that single photon emission can still be identified after quadruplicate the pulse frequency, as shown in Fig. 1b of the main text. This optimization is necessary, due to the drastic attenuation of the signal after passing through the high Q factor cavity, where the non radiative losses are dominant. The photon emission increment, together with the change to transmission configuration by etching the sample surface, enhances the signal in the EMCCD camera by a factor of approximately 16. A full experimental scheme, includ-

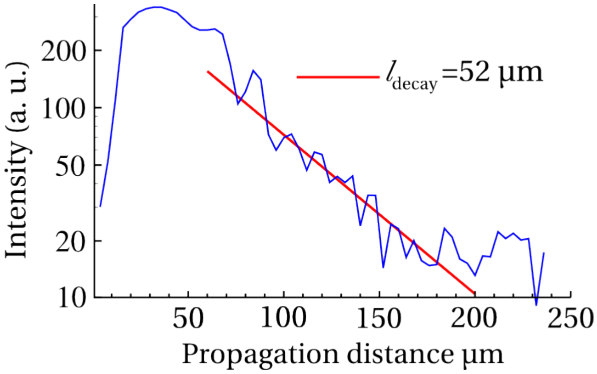

FIG. S2: Integrated intensity as a function of the propagation distance for figure 2c, plotted in a logarithmic scale. From an exponential fitting a decay distance of 52 μm, corresponding to a lifetime of 25 ps, is deduced.

ing the laser multiplexing is shown in Fig. S3. The three parts of the experiment mentioned in the main text (i) the generation of single photons, ii) the injection and propagation of single polaritons and iii) detection.) are denoted by dashed black lines in the figure.

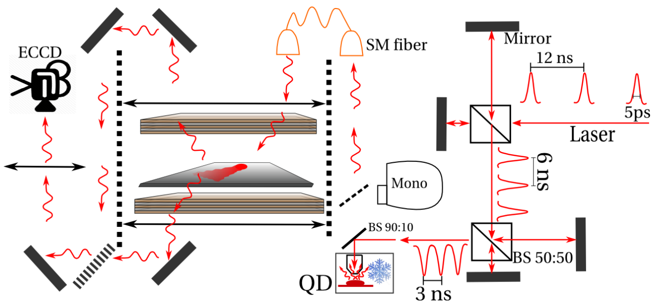

FIG. S3: Schematic experimental setup including laser multiplexing, reflection and transmission configurations.

PROPAGATION ACROSS A FINITE SIZE DEFECT

In figure S4 we show how the interference pattern is modified by the size of the defect. We compare the polariton in-plane wavelength $\lambda_{//}$ and the defect size r. The polariton in-plane wavelength is given by the polariton in-plane wavevector: $\lambda_{//} \approx 2\pi/k \approx 20\mu m$, given that $k \approx 0.28\mu m^{-1}$. In the first row, panel a) and b) show respectively the simulation and the experimental data for the case studied in this work, i.e. a point-like defect whose cross-section is much smaller than $\lambda_{//}$. In the second row, panel b) and c) show the simulation of the interference pattern for the cases $r = 1/4\lambda$ and $r = 1/2\lambda$. In the third row, panel e) and f) show the interference pattern for the cases $r = 3/4\lambda$ and $r = \lambda$. These interference pattern show that when the defect radius r is of the same order of magnitude as the in-plane polariton wavelength λ, a complex interference pattern arises, showing higher order interference effects, with several phase jumps across the interference fringes. These simulations clarify that the experimental fringes ahead of the defect cannot come from an overlap with the finite cross-section of the defect. In fact, the signature of a finite cross-section of the defect are not present in our experimental data. Similar results have also been shown in [2].

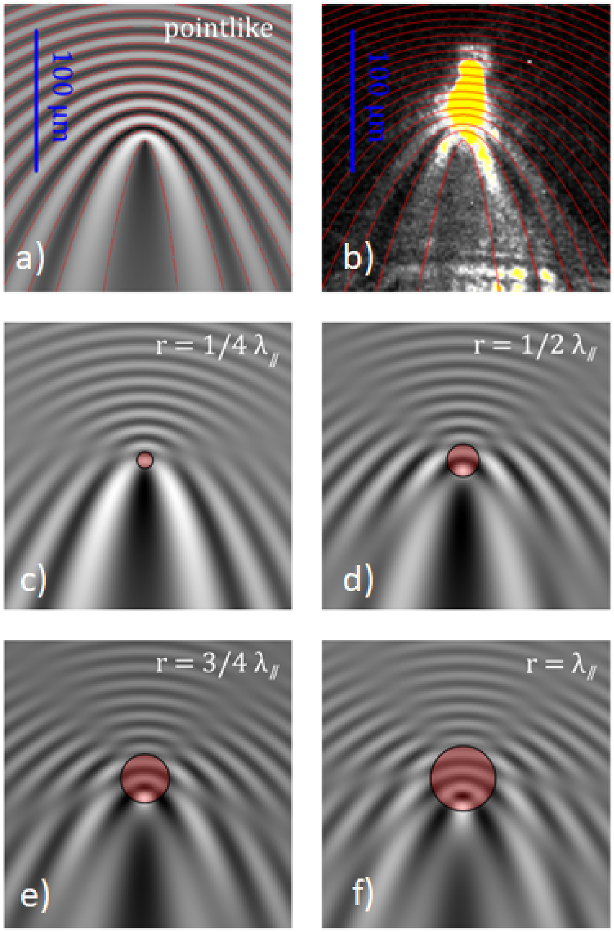

FIG. S4: Interference pattern of a plane wave scattered from a defect of different radius r (highlighted by the red circle). First row: simulation (left) and experimental data (right) for the case studied in our work, i.e., a point-like defect. Second and third rows: interference pattern for a defect of increasing size going from $r = 1/4\lambda_{//}$ to $r = \lambda_{//}$. In the case of an extended defect, higher order interferences appear. These effects are not present on our experimental data, strongly supporting our assumption of a point defect.

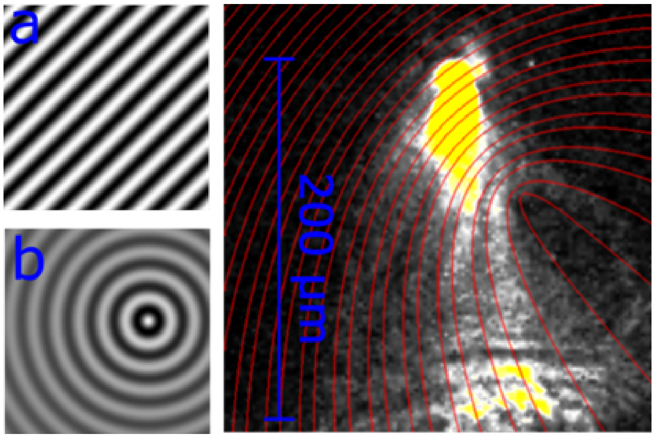

FIG. S5: a) Space distribution of the electric field of an incoming plane wave with a tilted in-plane momentum but same module as the used in Fig. 4 of the main text. b) Electric field distribution for circular wave, as it could be modeled for the light scattered from a punctual structural defect in the microcavity. c) Experimental density map from the Fig. 3c. A low fraction of the incoming light reaches and scatters against the defect, and an interference pattern can be noticed which is very weak and localized close around the defect. The parabolic fronts from the model of scattering from a punctual defect fits the fringes data well, confirming the suitability of the assumption. The numerical simulations are obtained by assuming an incoming polariton wave with an in-plane momentum $k \approx 0.28m^{-1}$, as in the experiment, but approximately tilted to 45° direction.

SINGLE POLARITON PROPAGATION IN THE CASE OF A PARTIAL SCATTERING

In this section we reproduce the pattern of the free propagating polariton (see figure 3c of the main text), where only a small fraction of the wave-packet is scattered by the defect, which is this time displaced around 60 m on the right side to the central propagation axis. The wavevector direction of the incoming plane wave has been modified to account for the spreading in the polariton packet propagation, whose momentum is locally tilted under the defect, see figure S5 a). The theoretical fitting to the barely visible interference pattern is displayed on the right side of Fig.S5. The good agreement of the model with the experimental data indicates once again the suitability of the assumption of punctual structureless defect. The specific tilted direction of the momentum is an approximation which is strictly valid only locally, close around and after the defect along its tilted line of sight, which is however also the area where the intensity of the circular wave is not negligible.

SECOND ORDER CORRELATION FUNCTION AT DIFFERENT REPETITION RATES

In order to increase the single photons flux arriving on the microcavity, we used a couple of Michelson and Morley interferometers. We obtained a 320 MHz pumping rate, resulting in 140 thousands single photons per second impinging on the microcavity. The low values of the second order correlation function in the three cases shown in figure S6 confirms that the system is always in the single photon regime.

* v.ardizzone85@gmail.com
† daniele.sanvitto@nanotec.cnr.it

[1] Alexey Kavokin, Jeremy J. Baumberg, Guillaume Malpuech, and Fabrice P. Laussy. *Microcavities*. Oxford Science, second edition, 2008.

[2] P. Cilibrizzi, H. Ohadi, T. Ostatnicky, A. Askitopoulos, W. Langbein, and P. Lagoudakis. Linear wave dynamics explains observations attributed to dark solitons in a polariton quantum fluid. *Phys. Rev. Lett.*, 113:103901, Sep 2014.

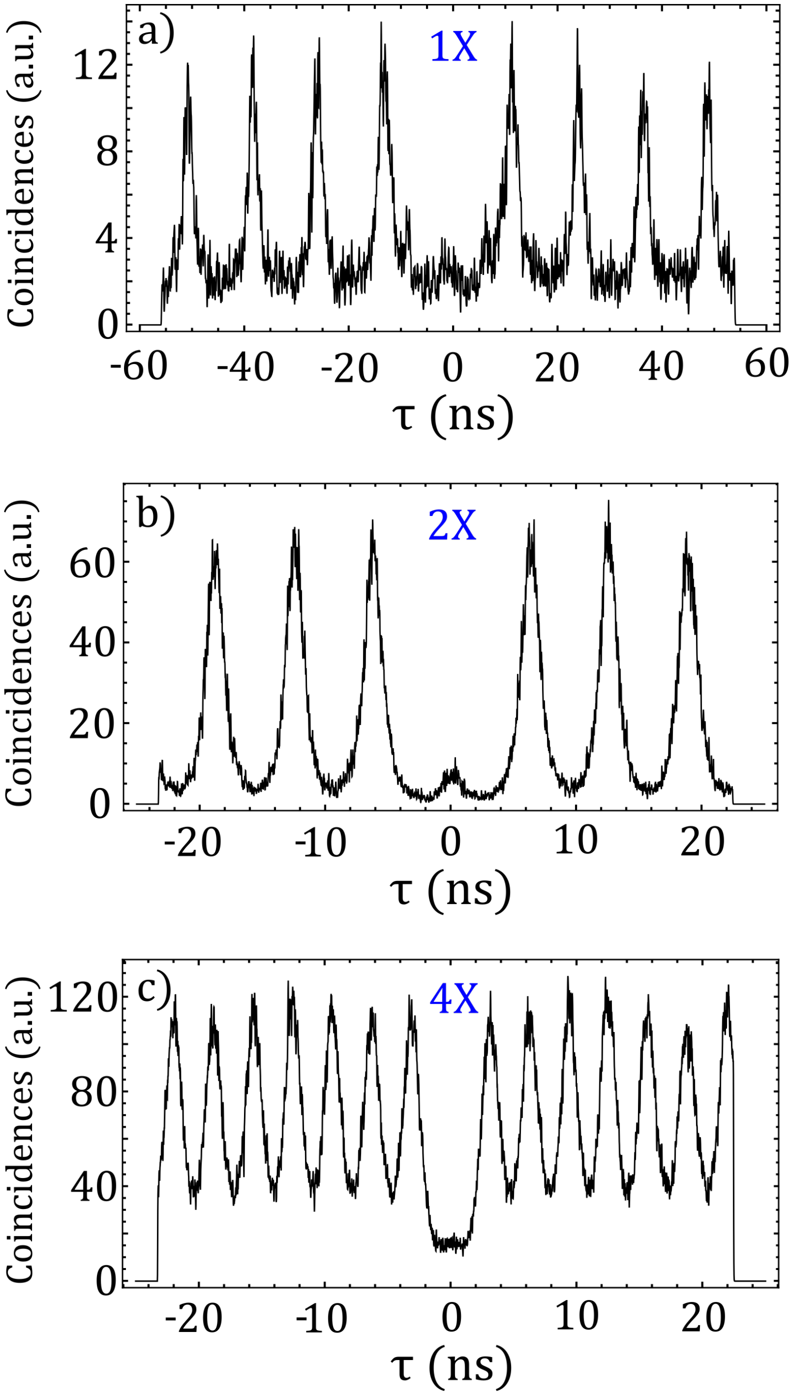

FIG. S6: Second order correlation function measured before any modification of the laser repetition rate (a), after its duplication with a Michelson and Morley interferometer (b) and after its quadruplication by mean of two cascaded interferometers (c). In every case, the data confirms the regime of single photons.
